# Enzyme-Assisted Fluorescence Biosensor Based on Circular Single-Stranded DNA Without Group Modification for MicroRNA Detection

**DOI:** 10.3390/bios14110527

**Published:** 2024-11-01

**Authors:** Xiaoxue Yin, Yazhen Liao, Feiyu Li, Jianbao Li, Jie Du

**Affiliations:** School of Materials Science and Engineering, Hainan University, Haikou 570228, China; 22220856010035@hainanu.edu.cn (X.Y.); 21220856000036@hainanu.edu.cn (Y.L.); 20213007198@hainanu.edu.cn (F.L.); 20213007169@hainanu.edu.cn (J.L.)

**Keywords:** duplex-specific nuclease, fluorescence reduction, GelRed, miR-34a

## Abstract

Fluorescent biosensor, which has the characteristics of high sensitivity, specificity, and low cost, can be directly detected in physiological fluids such as blood and serum. Therefore, the development of fluorescence sensor platforms for miRNA detection has a positive effect on the prevention and treatment of various diseases. In this paper, miR-34a was selected as a biological indicator of Alzheimer’s disease (AD). We designed a circular single-stranded DNA (CSSD) biosensor, which uses two unmodified single-stranded DNA (ssDNA) with complementary ends, DNAa and DNAb, to form CSSD by DNA sequence pairing to improve thermal stability and achieve signal amplification. At the same time, CSSD can react with miR-34a, and then the DNA of the DNA–RNA chain is hydrolyzed by duplex-specific nuclease (DSN enzyme). Finally, miR-34a is released to partake in the subsequent step, thus realizing cycle amplification. By evaluating the change in fluorescence signal under the optimized conditions, we discovered that this approach exhibits impressive sensitivity, with a detection threshold reaching as low as 0.36 nM. This surpasses the performance of numerous preceding miRNA detection biosensors. Furthermore, the system displays excellent detection capabilities even in intricate settings like serum, showcasing a strong ability to differentiate and choose effectively. In summary, this is a signal-off fluorescent biosensor, which realizes the purpose of double amplification of biosensor signal by using CSSD and enzyme assistance so that it can be used as a valuable tool for early diagnosis of diseases.

## 1. Introduction

Alzheimer’s disease (AD) is a secluded, age-related neurodegenerative disease, which is the most common form of dementia [1]. The elderly are the people mainly affected by AD, and the incidence of AD increases with the increase in life expectancy [2]. The incidence of AD is expected to double every five years after the age of 65 [3], and there is currently no cure [4]. In the absence of effective treatment, early diagnosis plays a vital role in improving the subsequent condition, and the diagnosis of Alzheimer’s disease is expensive, so it is imperative to create a novel and cost-effective diagnostic approach [5]. MicroRNA (miRNA) are a length of 19 to 25 nucleotides of non-coding RNA [6], and they are involved in many critical biological processes. As a result of miRNA in the important role of gene expression [7], their disorders are associated with the pathology of many diseases [8], such as liver, skin, skeletal muscle, cardiovascular, immune, neurological and inflammatory system diseases, as well as cancer [9]. This also means that the miRNA biomarkers can be used in various diseases [10]. In the past, miR-34a was regarded as a biomarker for malignant tumors and cancers [11,12,13]. However, recent studies have shown that miR-34a is related to brain neurophysiology and pathology [14], especially Alzheimer’s disease. Therefore, in addition to being a cancer marker [13], miR-34a is also expected to be a biomarker for AD [5,14]. Due to the extremely low abundance of biomarkers in cells, the development of sensitive platforms for the determination of biomarkers has broad research prospects [15]. Biosensors, including colorimetric, Raman scattering spectroscopy, electrochemical and fluorescent biosensors, etc., are characterized by high sensitivity, high specificity and high cost effectiveness, and can be directly detected in physiological fluids (blood, serum, etc.) [16]. Many researchers choose to use fluorescence detection biosensors to detect biomarkers [17], and their methods are widely used in medicine, food analysis, environmental and agricultural analysis, and industry [18,19,20]. DNA fluorescence biosensors transform the chemical energy of the tested biomaterial system into light energy. This categorization divides DNA fluorescence sensors into two groups: those with labeling and those without. Wang et al. designed a unique DNA detection method that involves the assembly of two functional nucleic acids to form a new nucleic acid structure upon exposure to the target DNA [21]. The nucleic acid structure represents a functional catalytic nucleic acid that can undergo substrate cleavage reactions in the presence of Mg^2+^. This leads to the cleavage of DNA labeled with fluorophore and cataplexy groups, resulting in a transition from low to high fluorescence intensity. Consequently, this method enables the quantitative detection of target DNA, with a sensor sensitivity of 0.01 pM and excellent selectivity.

Double-stranded specific nuclease (DSN) selectively degrades DNA in double-stranded DNA and DNA–RNA hybrids, has little activity against single-stranded nucleic acid molecules and double-stranded RNA, and can distinguish single-base mismatches well. The characteristics of DSN enzymes make them a promising option for signal amplification in DSN-based biosensors [22]. Liao et al. devised a nanochannel biosensor incorporating a DSN enzyme signal amplification technique [23]. In this system, the capture probe DNA is firmly attached to the surface of the nanochannel. Upon interaction with the target miRNA, the nanochannel undergoes hybridization, resulting in the formation of the DNA–RNA duplex. The DSN enzyme cleaved the probe DNA in the DNA–RNA double-stranded structure to extract the target miRNA. The target miRNA can then bind to other DNA probes, leading to signal amplification. GR serves as a secure and reliable intercalating dye, attaching to nucleic acid through electrostatic and charge interactions. It can directly replace the highly toxic ethidium bromide (EB), and its sensitivity is much higher than that of EB.

In this experiment, we designed an enzyme-assisted fluorescence biosensor without group modification. Based on the Watson–Crick base complementary pairing principle, the design had two single-stranded DNA (ssDNA) strands so that their terminals are complementary sequences, and there were two miR-34a binding sites on each DNA strand. The two ssDNA were hybridized to form circular single-stranded DNA (CSSD). GR intercalation dye was then added, which could select the DNA specifically and was fluorescent. The stronger the thermal stability of DNA, the stronger the fluorescence signal [24]. When the DSN enzyme is activated, it can cause the DNA strand in double-stranded DNA-RNA to be hydrolyzed, leading to the loss of the fluorescence signal if there is a specific miRNA that binds to CSSD. The target RNA chain and GR were released and they entered the next cycle. If no target miRNA was present, the fluorescence signal was always present. The sensitivity and specificity of the sensor were measured. This kind of biosensor provides a new idea for RNA detection.

## 2. Materials and Methods

### 2.1. Materials

The chemicals and reagents employed in the experiments are of analytical reagent grade and do not need any purification of the received pharmaceutical products. The DSN buffer was made up of a solution containing 50 mM Tris-HCl (pH 8.0), while the DSN master buffer was a mixture of 500 mM Tris-HCl (pH 8.0) and 50 mM MgCl_2_. DNA annealing buffer (5×, sterile solution, 0.1 M Tris-HC1, 0.1 M NaCl, 0.05 M EDTA) and PCR amplification buffer (10×, pH 8.3, 100 mM Tris-HCl buffer, 15 mM Mg^2+^, 500 mM KCl.) were purchased from Shanghai Yuanye Biotechnology Co., Ltd. (Shanghai, China); GR nucleic acid dye (10,000×) aqueous solution was purchased from Sangong Bioengineering Co., Ltd. (Shanghai, China); Tris-HCI buffer (pH 8.0) was purchased from Shanghai MacLean Biochemical Technology Co., Ltd. (Shanghai, China); DEPC-treated water was purchased from Beijing Solarbio Science & Technology Co., Ltd. (Beijing, China); glycerol solution (100%, sterile) was purchased from Beijing Ranjeco Technology Co., Ltd. (Beijing, China); DSN enzyme was purchased from Shenzhen Newbond Biotechnology Co., Ltd. (Shenzhen, China); and Fetal bovine serum (FBS) was purchased from Zhejiang Tianhang Biotechnology Co., Ltd. (Deqing, China).

The DNA utilized in this experiment, as shown in Table 1, was supplied by Shanghai Sangong Bioengineering Co., Ltd. (Shanghai, China). The lengths of DNAa and DNAb are 66 nt, and they contain two fragments complementary to miR-34a with a length of 22 nt. In addition, they have a complementary 9 nt fragment at each end. The resulting CSSD has a double chain length of 18 nt and a single chain length of 48 nt.

### 2.2. Apparatus

Fluorescence signal values were detected using the Shimadzu RF-6000 fluorescence spectrophotometer (Shimadzu, Kyoto, Japan). The experiment uses GR, an intercalated fluorescent dye that selectively binds DNA and RNA strands to produce a fluorescent signal. GR is attached to nucleic acids through electrostatic and charge interactions, and the fluorescence intensity of free GR is negligible. In the presence of ssDNA, GR was embedded into ssDNA to form GelRed-ssDNA complex with significant fluorescence intensity. Strong electrostatic interactions could stabilize the GelRed-ssDNA complex.

### 2.3. Preparation of Reagents

DNA was diluted to 10 µmol/L and miRNA was diluted to 20 µmol/L with DEPC treated water (enzyme free, sterile). Our choice of buffer for the experiment was the DNA annealing buffer (1×), stored in a refrigerator at 4 °C. The DSN buffer and the DSN master buffer both were stored at a temperature of 4 °C. Lyophilized DSN enzyme was added to DSN buffer at 5 µL per 10 U and an equal volume of 100% glycerol was added. The DSN enzyme, set at 1 U/µL, was stored at −20 °C for utilization.

### 2.4. Generation of CSSD

According to the previous work of Meng et al. [20], the ends of two ssDNA (DNAa, DNAb) are complementary sequences. First, 1 μM of DNAa and DNAb were added to the annealing buffer with 5 μL each, then heated at 95 °C for 2 min, and then cooled at room temperature for incubation. Finally, circular DNA was formed, and its concentration was 2.5 µmol/L.

### 2.5. Detection of Target miR-34a

According to the previous work of Sapia et al. [24,25], the experimental steps designed by us are as follows. Add 7.5 μL GR dye (3×) nucleic acid solution to CSSD solution and incubate at 25 °C for 15 min. After adding 2 μL of deionized water for 5 min at 95 °C, the mixture is cooled for 30 min at room temperature. Then, 0.5 μL DSN enzyme (1 U/μL) is added, and the DSN enzyme is inactivated at 45 °C for 1 h and 75 °C for 5 min. The measured fluorescence value is represented by F_0_. GR solution was added to CSSD solution and incubated at 25 °C for 15 min. Two μL miR-34a are added, incubated at 95 °C for 5 min, cooled for 30 min, and then the DSN enzyme is added. Then, the fluorescence value F is detected, and finally the fluorescence comparison result (F_0_-F) is obtained.

### 2.6. Testing miR-34a

The fluorescence signal value was measured by a fluorescence spectrophotometer with model RF-6000. The mixture used for the test consisted of 2 mL of ultra-pure water mixed with the experimental solution. The excitation wavelength used in this mixture was 270 nm, while the emission wavelength was 545 nm, and the target scan range was between 510 nm and 580 nm. The excitation frequency band was 5 nm and the emission frequency band was 10 nm.

## 3. Results and Discussion

### 3.1. Principle of miRNA Detection

**Figure 1:**
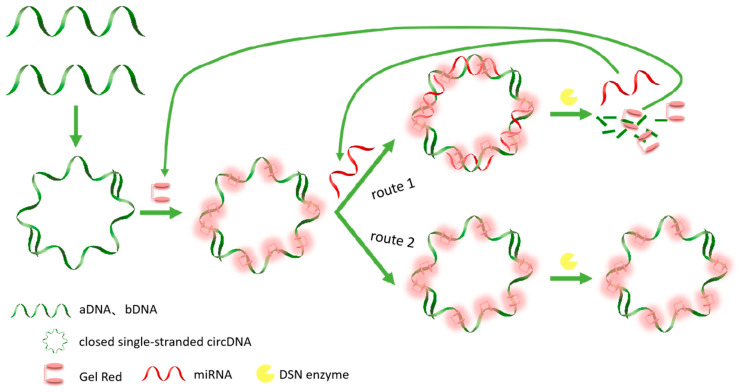
The principle of a biosensor based on CSSD amplification of enzyme-assisted fluorescent signals for miRNA detection.

The experimental design of the enzyme-assisted fluorescent biosensor detection principle of miR-34a is shown in Figure 1. In this study, we first designed two DNA single strands with complementary ends, DNAa and DNAb, which contain two complementary sequences of miR-34a, respectively. Therefore, hybridization of DNAa and DNAb produces CSSD with four miRNA binding sites. Since the fluorescence signal intensity of GR is enhanced with the increasing thermal stability of DNA, the fluorescence signal intensity can be improved after the two DNA single strands are combined to form a ring, and thus the fluorescence value F_0_ is obtained. Starting from the presence or absence of target detection miRNA, two routes can be obtained. Route one is that when the detection target exists, miR-34a carries out the hybridization reaction with circular DNA. At this time, under the action of the DSN enzyme, the DNA strand in the DNA–RNA hybrid strand is selectively hydrolyzed, miR-34a and GR dye are released, and the fluorescence signal value decreases. The released miR-34a can react with other unreacted circular DNA to achieve cyclic amplification. After the above reaction, the fluorescence value F was obtained after high temperature treatment and the DSN enzyme was inactivated. In route two, no RNA hybridizes with DNA due to the deletion of miR-34a. Even in the presence of DSN enzymes, single-stranded DNA cannot be hydrolyzed, so the fluorescence signal value remains unchanged. Finally, the fluorescence difference F_0_-F can be obtained by changing the fluorescence value, thereby determining the presence of miR-34a to be tested. Because the fluorescence values of the two lines are based on the GR dye, F_0_ and F are essentially the same.

### 3.2. Feasibility Study of the miR-34a Assay

**Figure 2:**
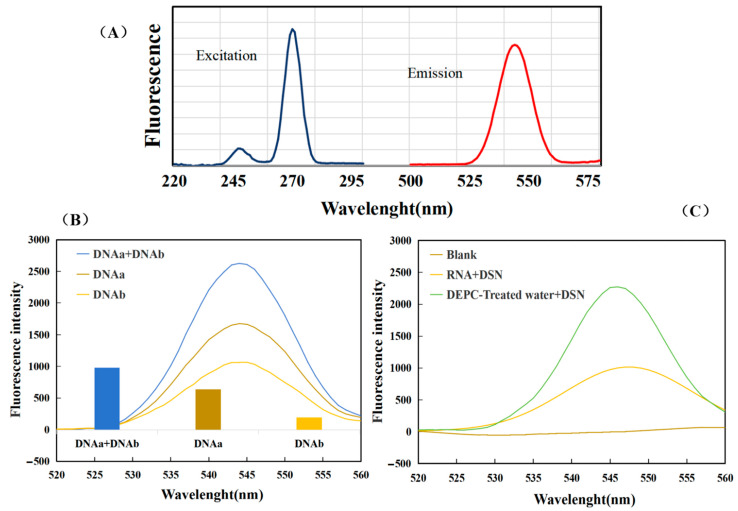
Feasibility analysis. (**A**) Excitation spectrum and emission spectrum of the sample to be measured. (**B**) The blue curve represents the fluorescence intensity with the addition of 5 µL DNAa and 5 µL DNAb, the brown curve represents the fluorescence intensity with the addition of only 10 µL DNAa, and the yellow curve represents the fluorescence intensity with the addition of only 10 µL DNAb. (**C**) The green curve shows the fluorescence signal curve with the addition of treated water and DSN enzyme, and the yellow curve shows the fluorescence signal curve with the addition of miRNA and DSN enzyme.

According to the excitation spectrum and emission spectrum of the sample to be tested, as shown in Figure 2A, we determined that the excitation wavelength for detection was 270 nm and the emission wavelength was 510–580 nm. In this experiment, in the presence of target miR-34a, two ssDNAs with complementary ends were combined to form CSSD. Oligo is one of the three most popular sequence design software in the world. It has powerful features, and its results are widely recognized. The structure of our designed single-stranded oligonucleotide was manually input into Oligo 7, and the Tm value was calculated using the nearest neighbor method. The Tm of DNAa and DNAb were measured at 77.1 °C and 76.1 °C, respectively. The Tm of PCR product CSSD was 96.9 °C, and the Tm of CSSD/RNA complex was 101.9 °C. The circular DNA strands were subjected to GR, a nucleic acid dye, to generate a fluorescent signal. Because GR fluorescence intensity is related to DNA thermostability, CSSD fluorescence intensity was higher than ssDNA fluorescence intensity. After that, under the action of the DSN enzyme, which only hydrolyzes the DNA strand in the DNA-RNA hybrid strand, the CSSD hybridized with the target strand miR-34a was hydrolyzed. The fluorescence signal generated by the binding of the DNA strand to the dye also disappeared. To confirm the viability of the experiment, we introduced the miR-34a target substance.

First, to verify whether the CSSD produced by ssDNA hybridization could really improve fluorescence intensity, three sets of comparison experiments were conducted. The experimental findings depicted in Figure 2B were obtained under the same conditions as those of our previous tests. The blue curve represents the fluorescence intensity with the addition of 5 µL DNAa and 5 µL DNAb, the brown curve represents the fluorescence intensity with the addition of only 10 µL DNAa, and the yellow curve represents the fluorescence intensity with the addition of only 10 µL DNAb. After the base complementary pairing, the fluorescence intensity of the addition of two ssDNAs was significantly greater than that of the addition of only one DNA, indicating that the fluorescence intensity was indeed improved. In addition, when the DNA concentration was the same, the fluorescence signal was significantly enhanced, which also indicated the formation of CSSD. The difference in fluorescence intensity of DNAa & DNAb, DNAa, and DNAb after enzyme digestion reaction is shown in Figure 2B. The value of DNAa & DNAb was significantly higher than that of single DNA, indicating that under the same concentration of RNA, the sensitivity of single DNA to RNA was significantly lower than that of CSSD.

To assess the viability of the sensor, we carried out experiments in parallel settings and acquired data, as shown in Figure 2C. When DEPC-treated water was added, the green curve shows its fluorescence intensity. When miRNA was added, the fluorescence intensity curve decreased significantly, indicating that miRNA was indeed hybridizing with DNA, and DNA was hydrolyzed by DSN enzymes, resulting in a decrease in fluorescence intensity. Therefore, this strategy can be used to detect miRNA.

### 3.3. Optimization of Experimental Conditions

In this experiment, the generation of CSSD and the hydrolysis of DSN enzyme were both critical. Hence, it was imperative to fine-tune the key experimental parameters influencing fluorescence intensity in order to enhance the biosensor’s efficacy. Here, we tested five key conditions, including the amount of GR dye, the incubation time of CSSD, the amount of DSN enzyme, the reaction temperature of the DSN enzyme, and the hydrolysis time of CSSD. Each group experiment had only one variable and conducted a series of three parallel experiments to reduce influence of random error. The other conditions were a DNAa (1 µM) dose of 2.5 µL, a DNAb (1 µM) dose of 2.5 µL, and a miR-34a (2 µM) dose of 1.5 µL.

#### 3.3.1. Optimization of Incubation Time of CSSD

**Figure 3:**
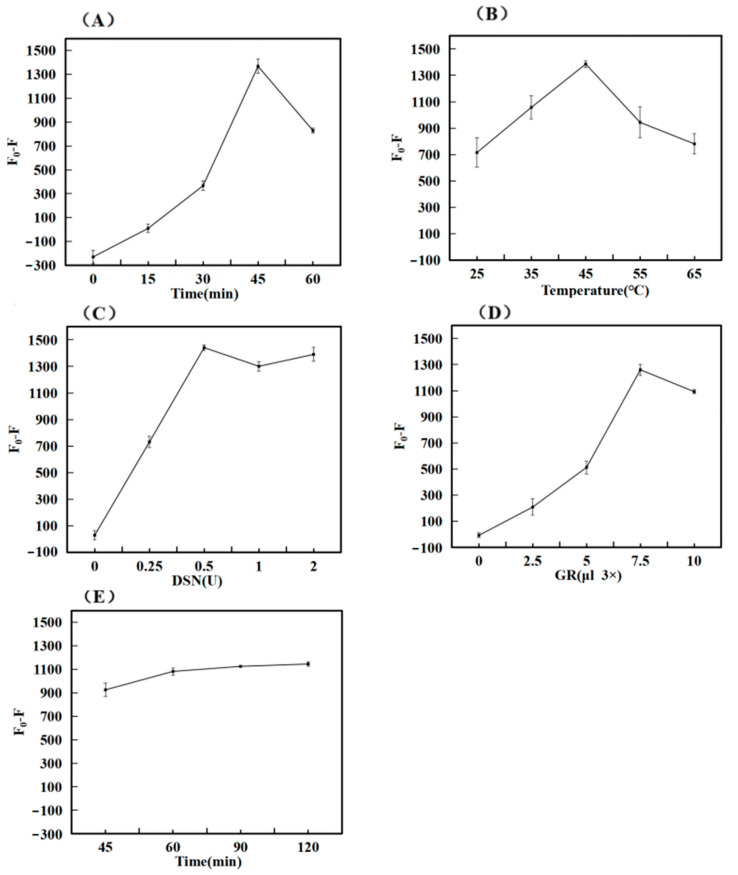
Optimization of experimental conditions. (**A**) Incubation Time of CSSD; (**B**) Reaction Temperature of DSN Enzyme; (**C**) Dosage of DSN Enzyme; (**D**) Dosage of GelRed Dye; (**E**) Time of DNA hydrolysis.

When the dosage of DNAa, DNAb, and miR-34a was determined, the amount of CSSD incubation was related to the level of fluorescence value F_0_, subsequently impacting the outcomes of successive experiments like reaction efficiency and signal output. The concentration of DNAa and DNAb configured and used in this experiment was 10 µM, and the incubation times of DNAa and DNAb were different under the same experimental conditions. A total of five experiments were set up, and each experiment was repeated three times. F_0_ was the fluorescence signal value for adding only DNA and GR, and F was the fluorescence value for adding RNA and DSN enzymes. As shown in Figure 3A, with the increase in incubation time, the fluorescence difference (F_0_-F) first increased and then decreased. The maximum value was reached at 45 min. The reason for it might have been the excessive time of incubation, which likely loosened some bound structures to gradually separate. Finally, the optimal time was chosen to be 45 min.

#### 3.3.2. Optimization of DSN Enzyme Reaction Temperature

Temperature is one of the important factors affecting the fluorescence intensity of hybridization. When the temperature drops too low, it can cause the reaction time to stretch or lead to incomplete reactions. Conversely, excessively high temperatures might hinder the activity of the DSN enzyme, thereby impacting the outcomes of the experiment. To address this, we designed five experiments to be tested at different reaction temperatures, and each test was repeated three times. As illustrated in Figure 3B, the difference in fluorescence (F_0_-F) shows an increasing trend between 25 °C and 45 °C, while it declines from 45 °C to 65 °C. This latter decrease could be attributed to the elevated temperature impairing enzyme activity. Considering both the cost-effectiveness and practicality of the biosensor, we determined that 45 °C is optimal for the reaction.

#### 3.3.3. Optimization of DSN Enzyme Dosage

The amount of DSN enzyme dosage was related to whether the DNA in the DNA–RNA hybridization chain could be completely hydrolyzed, thus affecting subsequent experiments. The concentration of DSN enzyme configured and used in this experiment was 1 U/µL. To establish the ideal concentration of the DSN enzyme, various amounts were introduced while maintaining consistent experimental conditions, ensuring that the volume of the test solution was standardized with the buffer solution. The outcomes of the tests are displayed in Figure 3C. As the quantity of DSN enzyme rose, the variation in fluorescence (F_0_-F) also increased, peaking at 0.5 U. However, beyond this point, additional increases in DSN enzyme dosage did not significantly alter the fluorescence intensity. To optimize resources, the DSN enzyme concentration was therefore maintained at 0.5 U for the following experimental adjustments.

#### 3.3.4. Optimization of GelRed Dye Dosage

The amount of GR affects the intensity of fluorescence signal detection. The concentration of GR dye used in the experiment was 3×. Under the same experimental conditions, GR dye with the same concentration but different amounts was added, and the volume of the tested solution was adjusted with ultra-pure water to be consistent. The experiment was repeated three times. After testing, the results obtained from the experiment are shown in Figure 3D. The difference in fluorescence (F_0_-F) rose as the dosage of GR dye escalated, peaking at 7.5 µL before tapering off with additional increments of the dye. This could be attributed to the possibility that at higher GR concentrations, there is less available binding space with DNA, which in turn leads to a reduction in the fluorescence signal. Therefore, the subsequent optimization experiment set the concentration of GR at 7.5 µL.

#### 3.3.5. Optimization of DNA Hydrolysis Time

The hydrolysis time of DNA is also the action time of the DSN enzyme. And the primary cause of the diminished fluorescence signal is the hydrolysis of DNA. When the hydrolysis time is too brief, it results in only a slight attenuation of the signal, ultimately compromising the sensor’s sensitivity. Conversely, if the duration is excessively lengthy, it leads to an inefficient use of time. In order to determine the ideal hydrolysis time of DNA, we changed the reaction time of the DSN enzyme and inactivated the enzyme with high temperature after reaching the expected time. Upon analyzing the results, we illustrated our findings in Figure 3E. We observed that the discrepancy in fluorescence grew over time, although the rate of increase began to slow down. Ultimately, we decided that a 60 min hydrolysis period was optimal for DNA.

### 3.4. Analysis of Performance of miR-34a Biosensors

In this study, we designed a DSN enzyme-assisted fluorescent biosensor platform for miR-34a detection. To investigate the detection performance of the sensor, under optimal experimental conditions, different concentrations of miR-34a were detected. Based on the above, the optimized experimental conditions were as follows: 0.5 U DSN enzyme, 1 h DNA incubation time, 45 °C DSN enzyme reaction temperature, and 7.5 µL GR dosage, the fluorescence signal value was the best result. Ten sets of experiments were conducted to measure the fluorescence intensity across various concentrations of miR-34a, with each set repeated thrice for accuracy.

**Figure 4:**
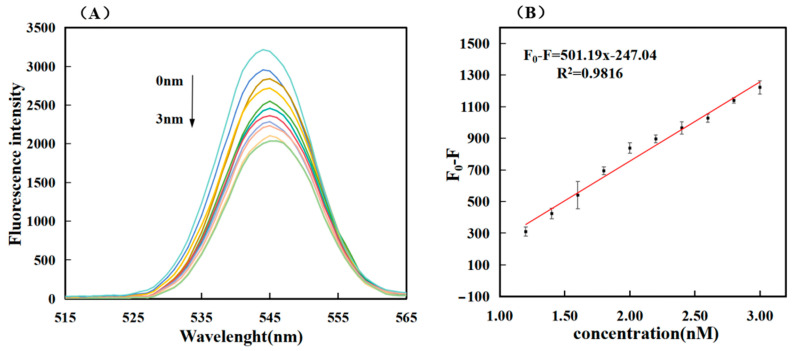
The fluorescence value of miR-34a was measured against the standard detection curve, and the concentrations of top to bottom substances in Figure (**A**) were 0 nM, 1.2 nM, 1.4 nM, 1.6 nM, 1.8 nM, 2.0 nM, 2.2 nM, 2.4 nM, 2.6 nM, 2.8 nM and 3.0 nM, respectively; Figure (**B**) Linear calibration curve of RNA and fluorescence intensity (correlation between RNA concentration and fluorescence signal strength). The error bars represent standard deviations obtained from from triplicate exper.

As shown in Figure 4A, this experiment is one in which the fluorescence value decreased with the increase in the target miRNA concentration. The standard detection curve in the sensitivity analysis was derived from the fluorescence value (F_0_) of the light blue curve in Figure 4A minus the curve (F) of different concentrations. Ten sets of different data (F_0_-F) were obtained, and the linear regression curves F_0_-F = 501.19C − 247.04 and R^2^ = 0.9816 were obtained through simple fitting, indicating the high feasibility of this experiment. The lowest detection limit (LOD) was determined by tripling the standard deviation (3σ) of the baseline signal. This was calculated using the formula 3σ/S, where σ represents the baseline signal’s standard deviation and S stands for the slope of the fitting line in Figure 4B. The detection limit was calculated to be 0.36 nM. The closer R^2^ was to one, the higher the accuracy of detecting the target miRNA within the detection range, and the better photobleaching observed in this experiment. As shown in Table 2 and the sensitivity of this work, our work is superior to many previous works on miRNA detection systems.

### 3.5. The Specificity of the Sensor

The experimental test of resistance to interference from other substances was also an important indicator of sensor performance. In order to see the influence of other miRNA sequences on the detection results, the signal values obtained from the detection of the target miR-34a, and other homologous miRNA were tested under the same optimized conditions. In theory, other non-targeted miRNAs cannot fully react with the designed DNA strand, and the efficiency of enzyme reactions will decrease. In order to verify whether the sensor constructed in this experiment was selective for the target miR-34a, we performed specific detection experiments using different miRNAs. These included miR-155, miR-192, miR-15a, miRNA-122, miR-660, and miRNA-21. Among them, the concentration of miR-34a was 3 nM, and the concentration of other detected substances was 15 nM.

**Figure 5:**
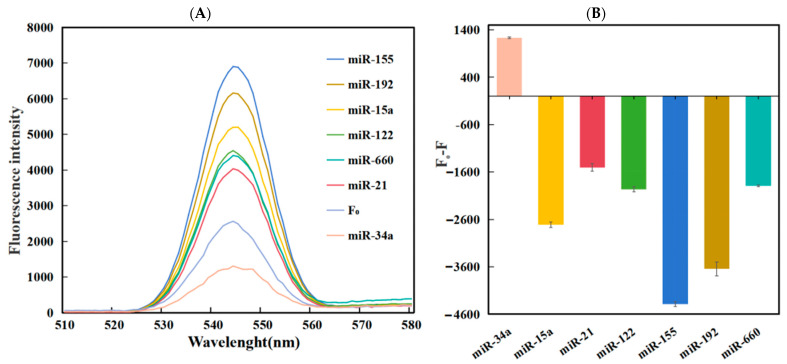
(**A**) Fluorescence spectrum plot of the relationship between miRNA and fluorescence intensity of different species; (**B**) Plot of different miRNAs of fluorescence intensity at the excitation wavelength of 545 nm.

The findings from the experiment are illustrated in Figure 5, where (A) displays the fluorescence spectra for different miRNA detection. Upon examination of the figure, it is clear that the fluorescence values of the remaining miRNA are quite high, whereas the fluorescence level of miR-34a is significantly lower. This suggests that in the experiments, other types of miRNA had difficulty binding to circular DNA and performing enzymatic reactions. The DNA was not hydrolyzed, but other RNAs were stained with GR dye, resulting in enhanced fluorescence intensity. As can be seen from the bar graph in Figure 5B, after three tests, the fluorescence intensity of the target miR-34a decreased, resulting in a positive fluorescence intensity difference, while the staining of other miRNAs increased the fluorescence intensity, resulting in a negative fluorescence intensity difference. Thus, the target RNA was very different from other miRNAs. In summary, this approach exhibits strong discriminatory capacity towards other similar miRNAs, showcasing commendable specificity and selectivity.

### 3.6. Detection of miR-34a in Complex Environments

To further verify whether our method was suitable for detection in complex environments, we recovered three different concentrations of the target RNA (1.5 nM, 2.0 nM, 2.5 nM) by adding different concentrations of the target DNA to the reaction to study the reaction in the amplification system. As shown in Table 3, Added (nM) refers to the actual added RNA content. The estimated RNA content, marked as Found (nM), was obtained by calculating the obtained fluorescence value. The ratio between the two is marked as Recovery (%), and RSD is the standard deviation of the recovery rate. The calculated recoveries ranged from 92.8% to 105.6%, and the relative standard deviation recoveries were 5.2%, 6.3%, and 5.5%. The recovery fluctuated within a small range of 100%, meeting the experimental requirements. A lower standard deviation suggests that the test results were more consistent with the expected results, pointing to greater accuracy in the method. The experimental findings indicate that the proposed detection method holds promise for detecting miR-34a in real, intricate biological samples.

## 4. Conclusions

In conclusion, we have constructed an enzyme-assisted fluorescent biosensor, leveraging CSSD for the detection of miRNA. This sensor employs the DSN enzyme to break down the CSSD that is hybridized with the target miR-34a. As a result, miR-34a is freed to engage in the next phase, facilitating cycle amplification. During this process, the DNA’s interaction with GR is disrupted, leading to a reduced fluorescence signal. Experimental findings demonstrated a clear distinction between the target miRNA and other RNA, illustrating strong selectivity and suitability for qualitative analysis. Notably, this method is capable of detecting miRNA at remarkably low concentrations, with a limit of detection as low as 0.36 nM and a linear detection range extending from 1.2 nM to 3 nM. Impressive outcomes were achieved even in intricate settings. This approach can also be adapted to identify various RNA biomarkers linked to illnesses by modifying the specified target recognition sequence. It offers considerable practicality and numerous potential applications in disease prevention and treatment, as well as for the quantitative measurement of RNA in medical research, paving the way for innovative studies.

## Figures and Tables

**Table 1:** Oligonucleotide sequences used in this experiment.

Name	Sequence (from 5′ to 3′)
miR-34a	5′-UGG CAG UGU CUU AGC UGG UUG U-3′
DNAa	5′-GCTGACTGCACAACCAGCTAAGACACTGCCATT TTACAACCAGCTAAGACACTGCCACGATCTCAC-3′
DNAb	5′-GCTAGAGTGACAACCAGCTAAGACACTGCCATT TTACAACCAGCTAAGACACTGCCACGACTGACG-3′
miR-34a	5′-UAGCUUAUCAGACUGAUGUUGA-3′
miR-192	5′-CUG ACC UAUGAA UUG ACA GCC-3′
miR-34a	5′-UGG AGU GUG ACA AUG GUG UUG-3′
miR-15a	5′-UAG CAG CAC AUA AUG GUU UGU G-3′
miR-660	5′-UAC CCA UUG CAU AUC GGA GGU GU G-3′
miR-155	5′-UUA AUG CUA AUC GUG AUA GGG GU-3′

**Table 2:** Comparison of some other sensors for miR-34a detection.

Detection Technique	Linear Range	LOD	Reference
PAMAM Dendrimer Modified Electrodes	0–7.5 µg/mL	135 nM	[26]
Ionic Liquid Modified Single-use Electrodes	2–10 µg/mL	125 nM	[27]
Ionic Liquid-Modified Graphite Electrodes	2–10 µg/mL	109 nM	[28]
The Chemical Activation of PGE Surface	0–2.5 µg/mL	72.5 nM	[29]
Graphene Oxide Modified Graphite Electrodes	142–568 nM	41.2 nM	[30]
Electropolymerization	5–80 µg/mL	28.4 nM	[31]
Enzyme-assisted fluorescence biosensor	1.2–3 nM	0.36 nM	This work
Gold Nanoflower @Graphene Quantum Dots Probe	0.4–4 fM	0.1 fM	[32]
Catalytic Hairpin Assembly &DNA Walker	200 pM–10 nM	43.72 pM	[33]
Gold Nanostructures	0.1–1000 nM	39 pM	[5]

**Table 3:** Recovery of miR-34a in FBS (*n* = 3).

Sample	Added (nM)	Found (nM)	Recovery (%)	RSD (%)
1	1.5	1.43	95.2	5.2%
		1.53	102.0	
		1.58	105.6	
2	2.0	2.10	105.0	6.3%
		1.86	92.8	
		1.93	96.6	
3	2.5	2.34	93.7	5.5%
		2.58	103.0	
		2.35	94.0	

## Data Availability

The raw data supporting the conclusions of this article will be made available by the authors on request.
